# Rural food security, subsistence agriculture, and seasonality

**DOI:** 10.1371/journal.pone.0186406

**Published:** 2017-10-19

**Authors:** Kibrom T. Sibhatu, Matin Qaim

**Affiliations:** 1 University of Goettingen, Department of Agricultural Economics and Rural Development, Platz der Goettinger Sieben 5, Goettingen, Germany; 2 University of Goettingen, Center of Biodiversity and Sustainable Land Use (CBL), Goettingen, Germany; SOAS, University of London, UNITED KINGDOM

## Abstract

Many of the world’s food-insecure and undernourished people are smallholder farmers in developing countries. This is especially true in Africa. There is an urgent need to make smallholder agriculture and food systems more nutrition-sensitive. African farm households are known to consume a sizeable part of what they produce at home. Less is known about how much subsistence agriculture actually contributes to household diets, and how this contribution changes seasonally. We use representative data from rural Ethiopia covering every month of one full year to address this knowledge gap. On average, subsistence production accounts for 58% of rural households’ calorie consumption, that is, 42% of the calories consumed are from purchased foods. Some seasonal variation occurs. During the lean season, purchased foods account for more than half of all calories consumed. But even during the main harvest and post-harvest season, purchased foods contribute more than one-third to total calorie consumption. Markets are even more important for dietary quality. During all seasons, purchased foods play a much larger role for dietary diversity than subsistence production. These findings suggest that strengthening rural markets needs to be a key element in strategies to improve food security and dietary quality in the African small-farm sector.

## Introduction

Despite significant progress in reducing global hunger over the last few decades, food insecurity and undernutrition remain serious problems in many countries [1–4]. Around 11% of the world’s population are chronically undernourished, meaning that these people do not have sufficient access to calories [4]. One-third of the global population suffers from micronutrient malnutrition, mainly due to insufficient intakes of vitamins and minerals to support a healthy life [1, 4]. Most food-insecure and undernourished people live in Asia and Africa. In sub-Saharan Africa, the number of undernourished people is even increasing [3]. Much of the food in Asia and Africa is produced by smallholder farmers [5], and, ironically, smallholder farmers are also those most affected by food insecurity [6–8]. Hence, the small farm sector is a crucial entry point for policy interventions to improve food security and nutrition. However, while agricultural interventions have a proven track record of increasing economic growth and reducing poverty, evidence about nutrition effects is relatively thin [9–12]. Many studies on the impacts of agricultural interventions have not rigorously analyzed effects on farm household diets and nutrition [9, 13].

With the discussion about the post-2015 development agenda and the United Nations’ Sustainable Development Goals, the need for making smallholder agriculture and food systems more nutrition-sensitive was prominently recognized [2, 9–15]. A key objective is to improve the diets of smallholder families in terms of both quantity and quality. But how exactly this can best be achieved remains elusive. Some suggestions focus primarily on increasing the diversity of foods produced on smallholder farms [16–20], while others focus more on improving smallholder access to markets [21–25].

Increasing production diversity on smallholder farms through introducing additional crop and livestock species can improve smallholder diets and nutrition through the subsistence pathway. It is well known that smallholder households typically consume a sizeable part of what they produce at home. A farm diversification strategy is also supported by ecologists, as this can help to maintain and increase agrobiodiversity [26–29]. On the other hand, most farm households also buy some of their food from the market, for which cash income is needed. Especially in sub-Saharan Africa, small farms typically already produce various crop and livestock species. In such situations, further diversification may potentially reduce cash income because gains from specialization cannot be realized [21, 30]. The best strategy to improve smallholder diets depends on the particular situation. One key question is how much of their food farm families actually obtain through the subsistence pathway and how much through market purchases. This is not yet sufficiently understood [31].

Recent studies have reported that market purchases contribute significantly to smallholder diets in sub-Saharan Africa [2, 21, 27, 30, 31]. Yet this evidence is based on data from cross-section surveys that were carried out in selected seasons of the year. Diets of poor rural households vary seasonally [32–35]. A number of studies have examined the effects of seasonal variations on dietary adequacy and nutritional outcomes in women and children [35–37]. We are not aware of any empirical research that has analyzed the seasonal contribution of subsistence production and market purchases to food security and dietary quality in smallholder farm households.

Here, we address this research gap with data from Ethiopia. The 2010/11 Household Consumption and Expenditure Survey (HCES) [38] is nationally representative for Ethiopia when appropriate sampling weights are used. We focus on rural areas, as we are interested in farming households. The survey contains data on all foods consumed by households collected every month over a period of one year (see Materials and Methods at the end of this article). HCES also includes data on the different sources of food, allowing us to analyze the seasonal role of subsistence production and market purchases for dietary adequacy and diversity. These monthly data from Ethiopia are fairly unique to analyze seasonality. In addition to this data advantage, Ethiopia is an interesting country for such analysis, because of widespread undernutrition and the pervasiveness of smallholder farming that is typical for sub-Saharan Africa [2, 8, 39–41].

## Results

### Farming and food security in ethiopia

The large majority of rural households in Ethiopia are involved in small-scale farming [39]. The average farm size in Ethiopia is less than two hectares [42]. Farming is highly diversified; the average farm in Ethiopia produces 10 different crop and livestock species [21], partly for subsistence purposes and partly for sale. Next to food crops–such as sorghum, teff, wheat, and maize–many farms grow cash crops, including coffee, tea, and sugarcane.

Agriculture in Ethiopia is mostly rainfed, depending on two rainfall seasons, spring *(belg)* and summer *(meher)* rains. Seasonal patterns vary by region, but in most regions the summer rains, which typically occur between July and August, are much more important than the spring rains [43]. The summer season accounts for over 95% of total crop production in Ethiopia [44]. In most regions, the summer season harvest occurs between September and November [43].

Based on the HCES data, we calculated a mean calorie availability of 2559 kcal per capita and day in rural households over the entire 12-months period. This is somewhat higher than the 2444 kcal reported by Hirvonen et al. [34] for rural Ethiopia using the same dataset. The divergence is due to differences in the way calorie consumption was calculated. Hirvonen et al. [34] added up the calorie consumption of all households and then divided by the total number of persons included in the sample, whereas we calculated calorie consumption for each household separately (Materials and Methods) and then took the average of all households included. Both methods have their pros and cons. As our intention is to disentangle household diets by food source, the household-level approach is more appropriate for this analysis.

Based on our calculations, around 12% of the households consume less than 1500 kcal, 41% consume between 1500 and 2500 kcal, and 47% consume more than 2500 kcal per capita and day, which is the average energy requirement for male adults with average physical activity [45]. These numbers point at widespread undernutrition in rural Ethiopia.

### Seasonal variation in calorie consumption

**Fig 1:**
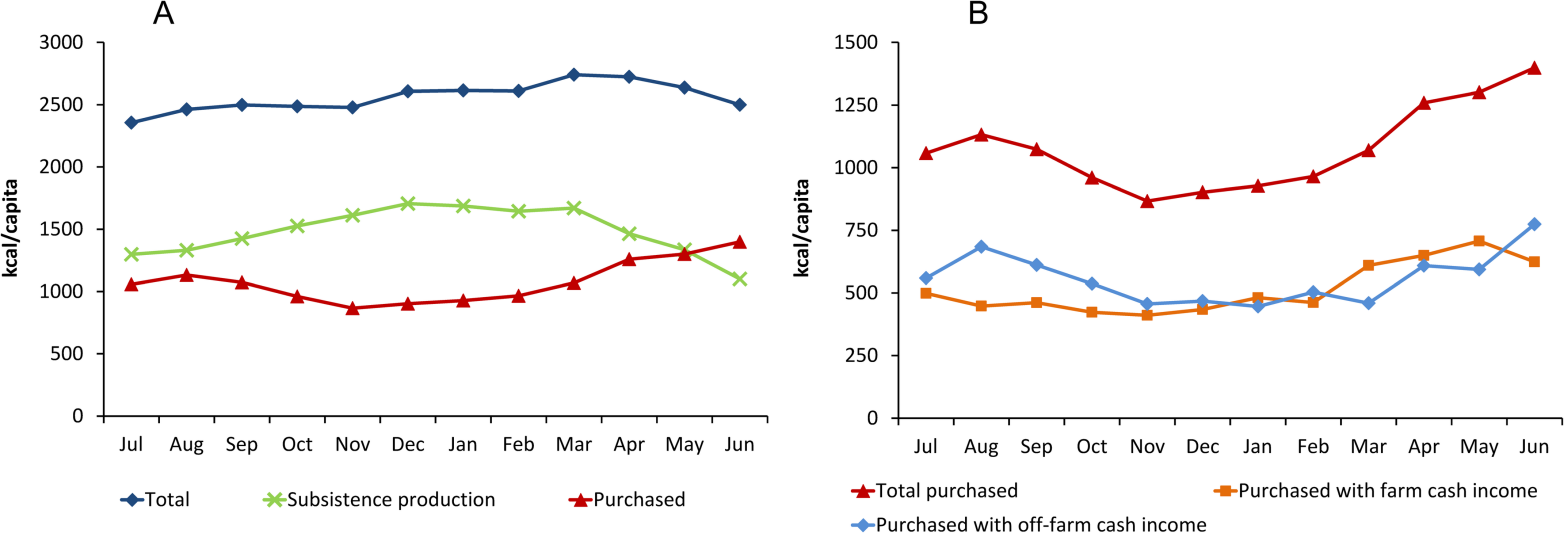
Seasonal variation in calorie consumption and role of different food sources. Data from rural households in Ethiopia (*n* = 10,322) collected between July 2010 and June 2011. (A) Breakdown of total average daily consumption by calories from subsistence production and calories from food purchased in the market. (B) Breakdown of calories from total purchased food by food purchased with farm cash income and with off-farm cash income.

Calorie consumption in rural Ethiopia varies considerably over the period of one year (Fig 1A). Consistent with the findings by Hirvonen et al. [34], the highest average per capita consumption of 2740 kcal is observed in March. This peak is probably less related to the agricultural seasons and more to the *Lent* fasting period of the Orthodox Christians, the largest religious group in Ethiopia. During the survey in 2010/11, the *Lent* fasting period occurred in March and early April. During this period, many people switched to vegan diets, entailing higher consumption of calorie-dense staple foods. The lowest per capita calorie consumption of 2350 kcal is observed in July, and this minimum is more related to the agricultural seasons. July is the lean season when the general availability of food is relatively low.

Fig 1A also shows a breakdown of calorie consumption by food source. Over the 12-months period, subsistence production accounts for 58% of rural households’ calorie consumption, meaning that 42% of all calories consumed are from foods that were purchased in the market. Previous studies with the same data and also with other datasets reported that subsistence production accounts for 40–50% of total food consumption in Ethiopia [2, 21, 46]. Those earlier studies looked at food consumption in terms of weight or monetary value, so they are not directly comparable with our results in terms of calorie shares. Moreover, some of the previous studies also included households in urban areas [2, 46], where own food production plays a less important role. When we include urban households in our analysis and use sampling weights to obtain representative results for Ethiopia as a whole, the share of subsistence production in total household calorie consumption decreases to 46% (S1 Fig).

We are not aware of previous research that has analyzed the seasonal variation of different sources of food, as we do here. Fig 1A shows that the role of subsistence production for calorie consumption in rural households varies seasonally: the average contribution is higher in the harvest and post-harvest season (September to February) and lower in the lean season (April to August). The highest share of subsistence in calorie consumption is observed in December (65%), the lowest in June (44%). Interesting to observe is that even at times when more food is available from own production, purchased food contributes more than one-third to total calorie consumption in rural households.

Another interesting feature of the HCES data is that households were also asked what source of cash income was used for buying the different food items consumed. In households where all income sources are pooled before expenditure decisions are made, such questions would be difficult to answer. However, in African farm households cash revenues from the various agricultural and non-agricultural economic activities are often controlled by different household members and earmarked for specific types of expenditures [47–50]. Fig 1B shows that cash income from farming and cash income from off-farm economic activities are both almost equally important for food purchases, with some seasonal variation. The role of food purchased with farm cash income increases between February and May, when the households’ own food stocks are gradually depleting. In contrast, the contribution of food purchased with off-farm income is highest between June and August. This is the time shortly before the next harvest, when own food stocks and agricultural incomes are lowest. Evidently, off-farm income, which is often less affected by seasonality than farm income, is an effective mechanism to smooth food consumption in rural households.

Over the entire 12-months period, food purchased with off-farm cash income contributes about 22% of total calorie consumption in rural households. The important role of off-farm income for rural food security was also stressed in previous studies referring to other countries in Africa [51]. However, in Ethiopia the rural off-farm sector is less developed than in many other African countries. On average, the share of off-farm income in total household income is less than 20% in rural Ethiopia [42], whereas it is above 30% in many other African countries [51, 52]. Off-farm income sources of rural households in Ethiopia include wage earnings, handicrafts, other small businesses, and remittances, among others.

### Seasonal variation in dietary diversity

**Fig 2:**
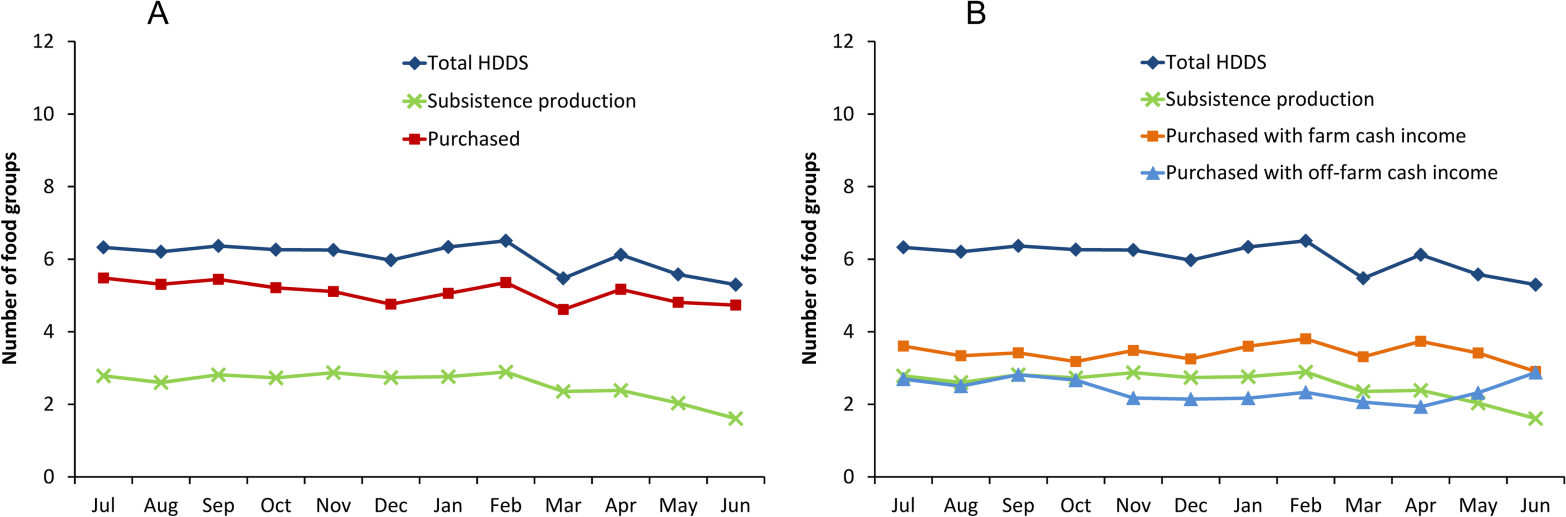
Seasonal variation in dietary diversity and role of different food sources. Data from rural households in Ethiopia (*n* = 10,322) collected between July 2010 and June 2011. HDDS, household dietary diversity score. A total of 12 food groups were considered. (A) Role of subsistence production and of purchased food. (B) Role of subsistence production and of food purchased with farm cash income and with off-farm cash income. The number of food groups consumed from different sources does not sum up to total HDDS, because the score does not increase when the same food group is consumed from different sources.

Beyond calorie consumption, dietary diversity is an important factor influencing nutritional outcomes. Hence, we have also analyzed the contribution of subsistence production and market purchases to dietary diversity. A common indicator of dietary diversity at the household level is the household dietary diversity score (HDDS), which measures the number of different food groups consumed by the household over a certain period of time (Materials and Methods). HDDS was shown to be positively correlated with micronutrient intakes and nutritional outcomes in many situations [53–56]. On average, farm households in Ethiopia consume around six different food groups (Fig 2). Over 80% of the food diversity is purchased in the market (Fig 2A), with farm cash income playing a larger role than off-farm cash income (Fig 2B). Only a relatively small part of the dietary diversity comes from subsistence production.

Looking at the seasonal patterns in Fig 2 more closely reveals that dietary diversity is lowest in March. Again, this is likely related to the *Lent* fasting period, when many Orthodox Christians switch to vegan diets (see above). Otherwise, seasonal differences in total HDDS are not very pronounced. Food diversity from subsistence production declines slightly during the lean season. This decline is partly compensated by an increase in diversity from food purchased with off-farm income. That markets are much more important for household dietary diversity than subsistence production throughout the entire year is an important finding. This is true in spite of the fact that farms in Ethiopia actually have quite diversified production patterns [21]. Producing a large number of crop species does not necessarily mean a large number of different food groups. For instance, sorghum, teff, wheat, and maize all belong to the same food group of cereals. But even if a larger number of food groups were produced by each farm and harvested once or twice per year, many food items are perishable and could not easily be stored for home consumption in other parts of the year.

**Fig 3:**
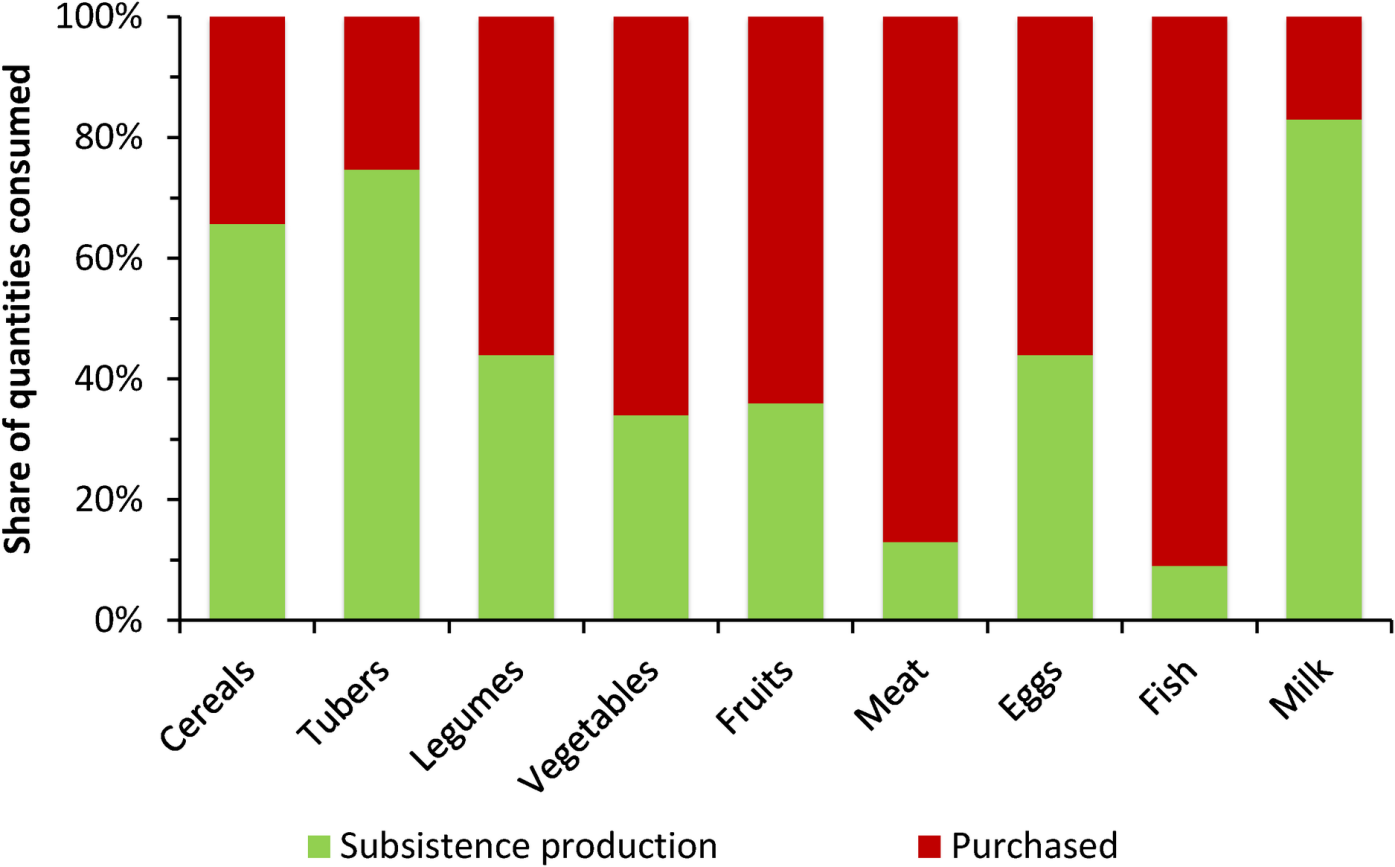
Role of subsistence production for the consumption of different food groups. Data from rural households in Ethiopia (*n* = 10,322) collected between July 2010 and June 2011. The food group classification is the same as for the calculation of the household dietary diversity score. Food groups that are completely purchased (sweets and sugars, oils, spices condiments, and beverages) are not shown.

Fig 3 confirms that the average role of subsistence production is more important for calorie-dense staple foods, such as cereals, than for more nutritious perishable foods, such as vegetables, fruits, and animal products. One exception is milk, for which the share of subsistence production is high. Unlike crops, milk production is less affected by seasonality. Moreover, recent research showed that milk markets and the milk processing sector in Ethiopia are poorly developed [57].

**Fig 4:**
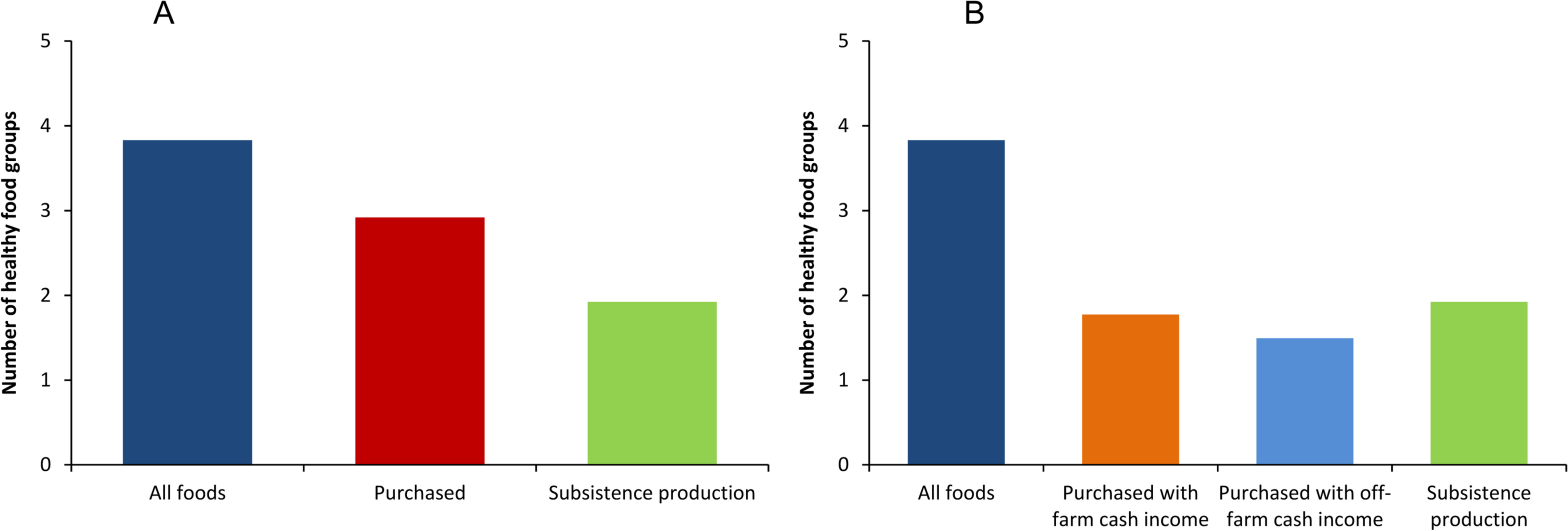
Number of healthy food groups consumed and role of different food sources. Data from rural households in Ethiopia (*n* = 10,322) collected between July 2010 and June 2011. A total of 9 food groups were considered. Numbers shown are averages over the 12-months period. (A) Role of subsistence production and of purchased food. (B) Role of subsistence production and of food purchased with farm cash income and with off-farm cash income. The number of food groups consumed from different sources does not sum up to the total from all foods, because the score does not increase when the same food group is consumed from different sources.

One drawback of HDDS from a dietary quality perspective is that it takes into account both healthy and unhealthy food groups. It has been argued that purchased food is often more processed, containing higher levels of sugar and fat and lower levels of micronutrients [15, 58, 59]. We tested whether subsistence production plays a more important role for rural household dietary diversity when only the more healthy food groups are included (Materials and Methods). Fig 4 reveals that the dominant role of markets persists also with this alternative indicator of dietary diversity. The same is true when we look at calories from non-staple foods as yet another indicator (S2 Fig). Throughout all seasons, food purchased from the market contributes more to dietary diversity than subsistence production.

### Variation by household nutrition status

**Fig 5:**
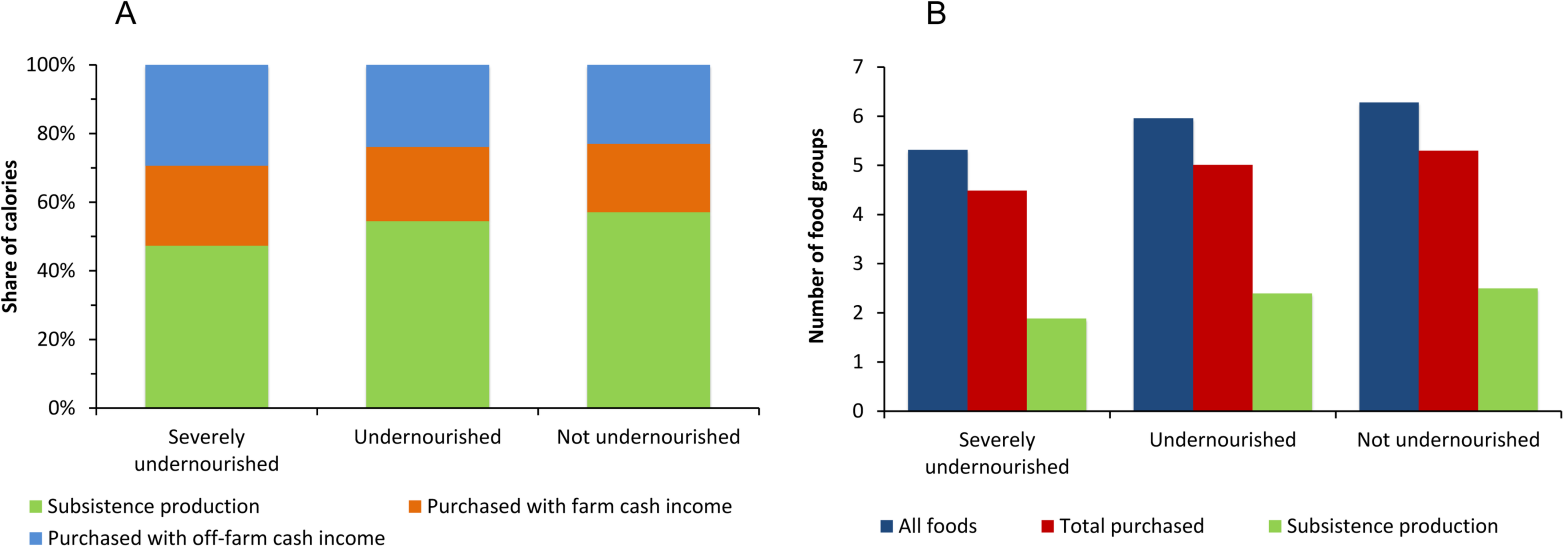
Role of different food sources by household nutrition status. Data from rural households in Ethiopia collected between July 2010 and June 2011. Household nutrition status is determined based on average daily per capita calorie consumption. Severely undernourished: <1500 kcal (*n* = 1184). Undernourished: 1500–2500 kcal (*n* = 4269). Not undernourished: >2500 kcal (*n* = 4869). (A) Contribution of different food sources to household calorie consumption. (B) Contribution of different food sources to household dietary diversity, measured in terms of the number of food groups consumed. A total of 12 food groups were considered. The number of food groups consumed from different sources does not sum up to the total from all foods, because the score does not increase when the same food group is consumed from different sources.

The results so far reflect averages for all rural households in Ethiopia. It is possible that for some households subsistence production is more important than for others. To analyze this further, we disaggregated households according to their nutrition status. For severely undernourished households, subsistence production contributes less to calorie consumption and dietary diversity than for households that are not undernourished (Fig 5A and 5B). This is perhaps unexpected, because the poorest and most undernourished rural households are often assumed to be more subsistence-oriented. Yet, severely undernourished households may also be the ones with the smallest farm sizes, which we cannot test because information about farm size is not included in the data. In any case, Fig 5 suggests that markets play an important role for all types of rural households in Ethiopia.

### Variation by agroecological zone

To analyze how the different sources of food vary by agroecological zone we ran a series of regression models (Materials and Methods). Estimation results are shown in Table 1. The base category in all models is the urban zone, which includes peri-urban areas within the range of bigger cities and towns. We also control for seasonality. For the calorie consumption models (columns 1–3), none of the agroecological zone coefficients is statistically significant. However, for the dietary diversity models, we observe negative and significant coefficients in columns (4) and (6). The negative coefficients in column (4) mean that households in the rural agroecologies have lower dietary diversity than households in the urban zone. For instance, households in the highland agroecology consume 0.71 fewer food groups than households in the urban zone. These effects are primarily driven by lower dietary diversity from purchased foods (column 6). Households in the rural agroecological zones are further away from urban centers and thus have poorer access to market infrastructure.

**Table 1:** Role of different food sources for household diets by agroecological zone.

	Household calorie consumption (kcal/capita)			Household dietary diversity score (number of food groups)		
	(1) All foods	(2) Subsistence production	(3) Purchased	(4) All foods	(5) Subsistence production	(6) Purchased
Highland agroecology	-123.99	0.13	-124.11	-0.71***	-0.35	-0.61***
	(161.04)	(159.602)	(158.98)	(0.18)	(0.23)	(0.22)
Moderate agroecology	-43.16	195.37	-238.53	-0.35**	-0.02	-0.23
	(159.14)	(157.83)	(156.86)	(0.17)	(0.23)	(0.21)
Lowland agroecology	-102.98	109.08	-212.06	-0.53***	-0.14	-0.46**
	(159.94)	(158.83)	(157.56)	(0.18)	(0.23)	(0.22)
Pre-growing season (March-May)	221.08***	-16.38	237.46***	-0.54***	-0.52***	-0.36***
	(38.22)	(39.11)	(36.18)	(0.05)	(0.06)	(0.06)
Growing season (June-August)	-61.12	-248.19***	187.07***	-0.19***	-0.30***	0.05
	(39.38)	(41.49)	(37.84)	(0.06)	(0.06)	(0.06)
Post-harvest season (December-February)	127.03***	159.03***	-32.01	-0.02	-0.01	-0.189***
	(35.38)	(37.05)	(32.95)	(0.05)	(0.06)	(0.06)
Constant	2554.68***	1381.42***	1173.26***	6.73***	2.90***	5.57***
	(160.62)	(158.56)	(157.64)	(0.18)	(0.23)	(0.21)
Observations	10322	10322	10322	10322	10322	10322
R^2^	0.01	0.02	0.02	0.03	0.02	0.02

Estimation results from linear ordinary least squares regressions. Sampling weights provided by the Ethiopian Central Statistical Agency were used. Estimation coefficients are shown with robust standard errors in parentheses. All explanatory variables are dummies. The base category for the agroecological zones is urban (within the range of bigger cities and towns). The base category for the seasons is the harvest season (September-November).

** Significant at the 5% level.

*** Significant at the 1% level.

The lower part of Table 1 shows differences by agricultural season, further underlining the results above. In 2010/11, total calorie consumption was highest in the pre-growing season (column 1), due to the Christian Orthodox fasting period that occurred in March and early April. During this fasting period, many households switched to vegan diets, with reduced dietary diversity (column 4) and a stronger focus on calorie-dense staple foods. In addition, higher calorie consumption was observed during the post-harvest period, mainly due to a larger role of subsistence production. Calories from subsistence production decrease during the growing season (column 2), but this decrease is counteracted by higher calorie consumption from purchased foods (column 3). Dietary diversity also decreases somewhat during the growing season (column 4), mainly due to lower availability from subsistence production (column 5). This decrease cannot be fully compensated through market purchases (column 6), which is possibly due to market price increases during this lean season. African food markets are often not sufficiently efficient and integrated to prevent considerable seasonal price fluctuation [60–62].

## Discussion

Undernutrition is still widespread among rural households in Africa. These households do not only suffer from insufficient calorie intakes, but also from low dietary diversity leading to multiple micronutrient deficiencies with severe negative health consequences. Hence, improving diets in terms of both quantity and quality needs to have high policy priority. But how can this be achieved? Most rural households in Africa are semi-subsistent farmers, meaning that they produce a certain part of the food they consume themselves. However, our results with representative data from rural Ethiopia show that markets are equally important for the diets of these households. On average, subsistence production accounts for 58% of rural households’ calorie consumption, that is, 42% of the calories consumed are sourced from the market. Some seasonal variation occurs. During the lean season, purchased foods account for more than half of all calories consumed. But even during the main harvest and post-harvest season, purchased foods contribute more than one-third to total calorie consumption. Markets are even more important for dietary quality. Purchased foods make up over 80% of the dietary diversity consumed, which is true in all seasons and for all types of households, including undernourished and sufficiently nourished ones.

The exact numbers from Ethiopia should not be extrapolated to other countries in Africa. However, Ethiopia’s small-farm sector is known for its relatively high subsistence orientation [24, 42, 44]. Indeed, a recent paper that reviewed data from various developing countries showed that Ethiopia is one of the countries with the highest share of subsistence production in household food consumption [2]. Against this background, the finding that markets play an important role for the diets of rural households is not specific to Ethiopia but also holds elsewhere. This has been confirmed by a few studies that have used cross-section data from other countries in Africa [21, 22, 25, 27, 31]. We are not aware of previous research that has analyzed seasonal patterns of subsistence production and market purchases, as we have done here for Ethiopia.

Our results clearly suggest that strengthening market access and thus opportunities to generate cash income should be a key element in strategies to improve diets and nutrition in the small-farm sector. Subsistence production cannot provide the dietary diversity that is needed for healthy nutrition throughout the year. African farm households typically already produce a large number of different crop and livestock species [21, 30]. Promoting further farm diversification through the introduction of additional species may reduce opportunities to generate cash income and can thus even have negative nutritional effects. This does not mean that farm diversity is bad and that all farms should specialize to produce only one or two different crop or livestock species. A certain level of farm diversity promotes environmental sustainability and is also needed for households to better cope with risk. But beyond a certain point, the marginal benefits of diversity decrease, whereas the marginal costs in terms of foregone cash incomes increase. Cash incomes are not only generated from agricultural sales. Many farm households derive a certain part of their total income from off-farm economic activities. Our results suggest that off-farm income is also important for regular food purchases, especially to smooth consumption during the lean season.

One aspect that needs some further reflection is that markets can be quite different and often do not function well in rural areas of Africa [57, 60–62]. Inefficiencies and high seasonal price fluctuations occur through poor infrastructure, weak institutions, and other types of market and government failures. Such constraints need to be addressed. Without this in mind, a push towards higher levels of market orientation can be counterproductive for improving diets in rural households. Another aspect worth highlighting is that diversifying farm and household production is not necessarily in contradiction to a market approach. However, rather than fostering subsistence, diversification strategies should keep market developments in mind. If farm diversification responds to market incentives and builds on comparative advantage, it can not only improve dietary quality through the subsistence pathway but can also help to generate cash income and add to the diversity of food supplies in local markets.

How can market functioning and market access for rural households in Africa be strengthened? Improved roads and market infrastructure clearly play important roles. In addition, various other components matter as well, including improved transport, storing and processing facilities, access to credit, information, and modern production technologies, as well as transparent quality grading systems, among others. Historically, food security policies in Africa were often primarily concerned about improving the production and marketing of cereals and other starchy staple foods [11]. This has contributed to a market bias against more nutritious foods such as fruits, vegetables, pulses, and animal products. Overcoming such biases and promoting developments in previously neglected foods could help to create new market and price incentives for smallholder producers. This would help to raise cash income opportunities and make smallholder food systems more nutrition-sensitive.

## Materials and methods

### Household survey data

Data for this analysis come from the 2010/11 Household Consumption and Expenditure Survey (HCES) in Ethiopia collected by the country’s Central Statistical Agency [38]. The survey covered the entire country, except for some nomadic pastoralists in the eastern regions. A multi-stage stratified random sampling approach was used to select households in rural and urban areas of the country. Details about the sampling procedure are described by the International Household Survey Network (IHSN) [63]. When sampling weights are used, the HCES is representative at national level [38]. The total dataset includes observations from 27,835 households: 10,322 from rural and 17,513 from urban areas. In this study, we used the subsample of 10,322 rural households, as we are interested in the role of own agricultural production for household food consumption. In urban areas, own agricultural production beyond small kitchen gardens is rare. For all calculations, we used appropriate sampling weights, so the results are representative for rural Ethiopia. In a robustness check, we tested how the main results look like when the entire sample of rural and urban households is used (S1 Fig).

HCES focuses on the consumption profiles of households and has little information on the agricultural production part. Data on farm size, types of crops grown, types of technologies used etc. are not included. For the general descriptions of the farming situation, we used other literature sources as cited in the text. In the food consumption module of the HCES questionnaire, respondents were asked to provide details of all food items consumed over a period of one week (7 days). To minimize recall errors, each household was visited twice during the survey week [38]. The questionnaire included a list of 275 food items that was compiled from detailed household diaries prior to the start of the actual survey. Respondents were asked to specify the quantities of each food item consumed by all household members. These quantities were recorded by source, namely (1) own production or collection (subsistence), (2) purchased with farm income, and (3) purchased with off-farm income. If a household consumed the same food item from different sources, the quantity from each source was recorded separately.

To capture seasonality in food consumption, data collection for the HCES was extended over the period of an entire year (July 2010 to June 2011). Every month, a pre-defined number of households in the different regions were visited, providing nationally representative data for each month. We used these seasonal food consumption data to calculate household calorie consumption and dietary diversity.

### Calculation of calorie consumption

We measure food availability in each household in terms of the number of calories consumed per capita and day, which is a common approach to assess food security [31, 45, 64]. Food quantities consumed were converted to calories using calorie conversion tables for Ethiopia [65]. Calorie consumption per capita and day (*CC*_*cd*_) was then calculated as:

$$CC_{cd}=\frac{C_{s}+C_{fi}+C_{oi}}{R\times N_{hh}}$$

where *C*_*s*_ is total weekly calorie consumption from subsistence production, *C*_*fi*_ is total weekly calorie consumption from food purchased with farm cash income, *C*_*oi*_ is total weekly calorie consumption from food purchased with off-farm cash income, *R* is the food consumption recall period (7 days), and *N*_*hh*_ is the number of members living in the household.

We also used *CC*_*cd*_ to classify households into three nutrition status groups. The thresholds used are the same as those used by Frelat et al. in a recent study [31]. Households with *CC*_*cd*_ < 1500 kcal are classified as severely undernourished (*n* = 1184); households with 1500 *kcal* ≤ *CC*_*cd*_ ≤ 2500 are classified as undernourished (*n* = 4269); and households with *CC*_*cd*_ > 2500 kcal (requirement of a male adult with average physical activity [45]) are classified as not undernourished (*n* = 4869).

### Calculation of dietary diversity

The household dietary diversity score (HDDS) is a common indicator of access to food and dietary diversity at the household level [53–56]. HDDS measures the number of food groups consumed by the household, categorizing food items into the following twelve groups [55]: cereals; white tubers and roots; legumes, legume products, nuts, and seeds; vegetables and vegetable products; fruits; meat; eggs; fish and fish products; milk and milk products; sweets, sugars, and syrups; oils and fats; and spices, condiments, and beverages. In our calculations of HDDS, we used these twelve food groups considering all food items consumed by the households during the 7-day recall period. As for calories, we also differentiate between food items consumed from subsistence production, from purchases with farm cash income, and from purchases with off-farm cash income. However, unlike for calories, the number of food groups consumed from the different sources does not sum up to the total. The reason is that the number of food groups does not increase when the same food group is consumed from different sources.

In the robustness check in Fig 4, we used an alternative food group classification. Instead of including all 12 food groups in the calculations, we excluded the last three (sweets, sugars, and syrups; oils and fats; and spices, condiments, and beverages), because these add little in terms of nutritional quality, especially with a view to micronutrient consumption [55]. The remaining 9 “healthy food groups” were used to calculate an alternative dietary diversity score, which is considered a better indicator of dietary quality [21].

One drawback of dietary diversity scores in general is that food groups are counted regardless of the actual quantity of food items consumed. That is, seasonal reductions in the quantity of certain food groups consumed will not be reflected in the dietary diversity score, unless households completely abandon a food group. An alternative indicator of dietary diversity that accounts for food quantity is the consumption of calories from non-staple foods [51, 66]. More calories from non-staple foods point at higher levels of diversity and better-quality diets. This alternative indicator was used for the results shown in S2 Fig.

### Regression models to analyze differences by agroecological zone

To analyze heterogeneity in household diets by agroecological zone we estimated a series of regression models of the following type:

$$D_{i}=\alpha +\beta^{\prime }{AEZ}_{i}+\gamma^{\prime }S_{i}+\varepsilon_{i}$$

where *D*_*i*_ is the dietary indicator of household *i* (calorie consumption or dietary diversity score), *AEZ*_*i*_ is the agroecological zone in which household *i* is located, *S*_*i*_ refers to the season when the data from household *i* were collected, and *ε*_*i*_ is a random error term. We estimated six models, each with a different dependent variable, *D*_*i*_. Three models refer to calorie consumption, and the other three to dietary diversity (number of food groups consumed), in both cases differentiating between (1) all foods, (2) subsistence production, and (3) purchased from the market. All models were estimated with an ordinary least squares estimator, using robust standard errors to control for heteroscedasticity.

In terms of agroecological zones (*AEZ*), the HCES differentiates between the following three zones: highland (*Dega*), moderate (*Woina Dega*), and lowland (*Kola*) [38]. This classification is based on temperature and altitude [67]. A fourth zone in the HCES classification is the urban zone. While in this study we concentrate on rural households, a few of these households are located in peri-urban environments, thus falling into the urban zone for this agroecological classification. A breakdown of the rural household sample by agroecological zone is provided in S3 Fig. In the regression models, we included dummy variables for the three agroecological zones and used the urban zone as the base category.

In terms of seasons (*S*), we used the following classification [43]: pre-growing season (March-May), growing season (June-August), harvest season (September-November), and post-harvest season (December-February). A breakdown of the sample by season is provided in S4 Fig. In the regression models, we included three dummy variables for pre-growing season, growing season, and post-harvest season, using the harvest season as the base category.

## Supporting information

S1 Fig Calorie consumption and role of subsistence in rural and urban households combined. (PDF)

S2 Fig Seasonal variation in calorie consumption from non-staple foods. (PDF)

S3 Fig Distribution of rural household sample by agroecological zone. (PDF)

S4 Fig Distribution of rural household sample by agricultural season. (PDF)
