# Supplementary material for: Rural food security, subsistence agriculture, and seasonality
Source: PLoS One. 2017 Oct 19;12(10):e0186406. doi: 10.1371/journal.pone.0186406 (PMC5648179; doi:10.1371/journal.pone.0186406)
Supplement: S1 Fig — (PDF) [file pone.0186406.s001.pdf]

S1 Fig. Calorie consumption and role of subsistence in rural and urban households combined.

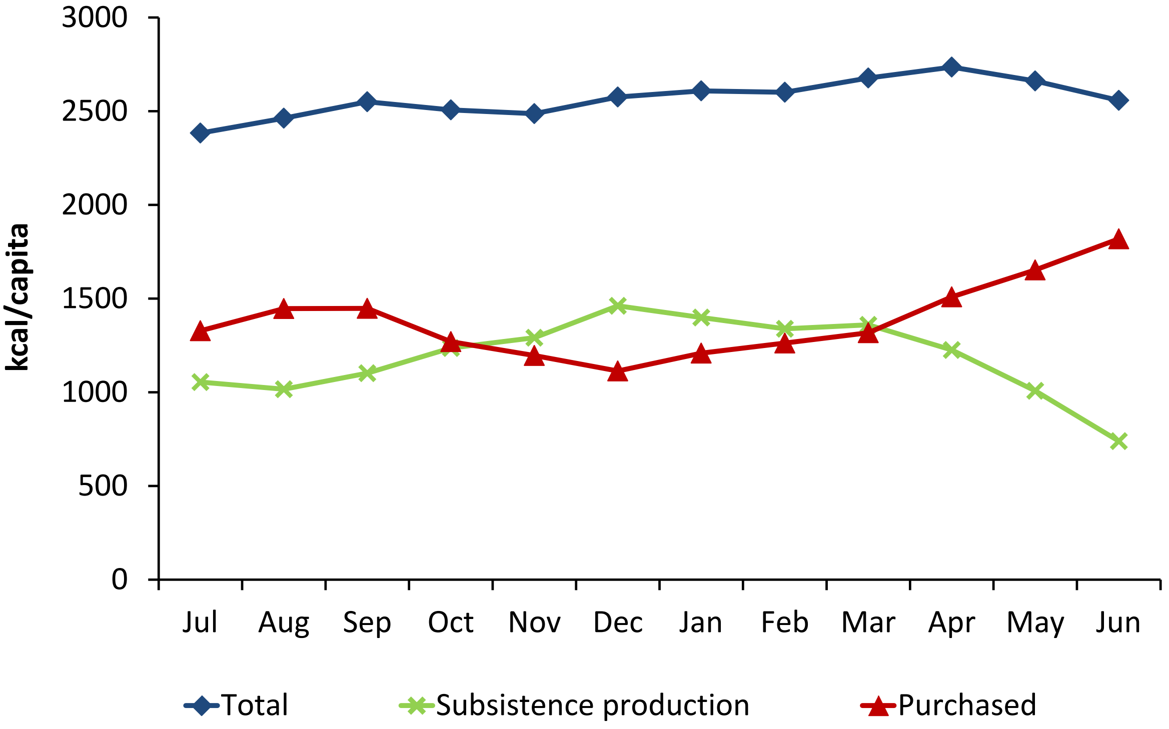

Data from rural and urban households in Ethiopia (*n*=27,835) collected between July 2010 and June 2011. Breakdown of total average daily calorie consumption by calories from subsistence production and calories from food purchased in the market.
