# Supplementary material for: Rural food security, subsistence agriculture, and seasonality
Source: PLoS One. 2017 Oct 19;12(10):e0186406. doi: 10.1371/journal.pone.0186406 (PMC5648179; doi:10.1371/journal.pone.0186406)
Supplement: S2 Fig — (PDF) [file pone.0186406.s002.pdf]

S2 Fig. Seasonal variation in calorie consumption from non-staple foods.

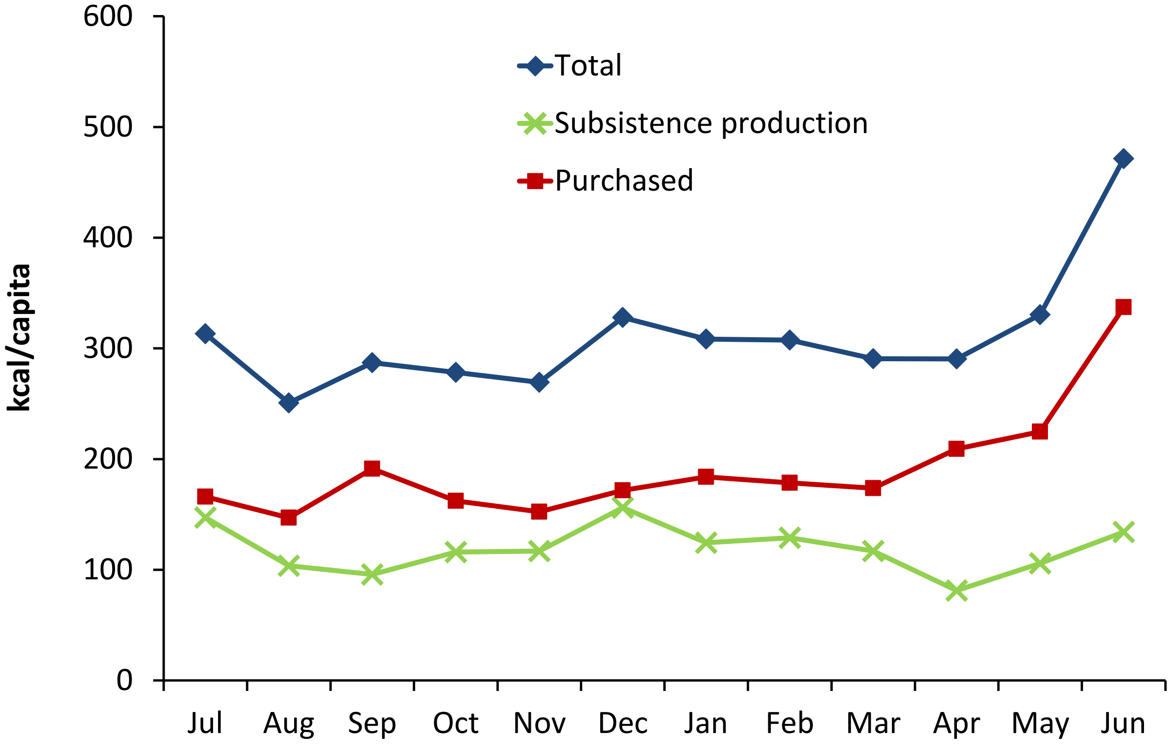

Data from rural households in Ethiopia (*n*=10,322) collected between July 2010 and June 2011. Calories from non-staple foods include legumes, nuts, seeds, fruits, vegetables, and all animal products.
