# Supplementary material for: Rural food security, subsistence agriculture, and seasonality
Source: PLoS One. 2017 Oct 19;12(10):e0186406. doi: 10.1371/journal.pone.0186406 (PMC5648179; doi:10.1371/journal.pone.0186406)
Supplement: S3 Fig — (PDF) [file pone.0186406.s003.pdf]

S3 Fig. Distribution of rural household sample by agroecological zone

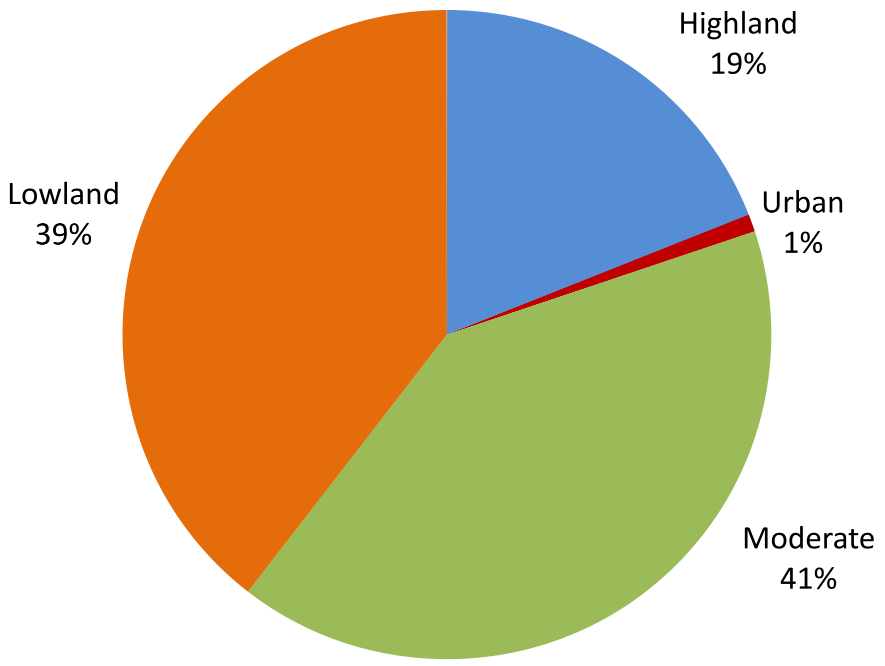

The total sample size of rural households in the 2010/11 Household Consumption and Expenditure Survey (HCES) in Ethiopia was 10,322.
